# Convergence analysis of Suzuki’s generalized nonexpansive mappings using the Picard–Abbas iteration process

**DOI:** 10.1371/journal.pone.0334440

**Published:** 2025-10-15

**Authors:** Bashir Nawaz, Krzysztof Gdawiec, Kifayat Ullah, Maha Noorwali, Maggie Aphane

**Affiliations:** 1 Department of Mathematics, University of Lakki Marwat, Lakki Marwat, Khyber Pakhtunkhwa, Pakistan; 2 Institute of Computer Science, University of Silesia, Bedzinska, Sosnowiec, Poland; 3 Department of Mathematics, King Abdulaziz University, Jeddah, Saudi Arabia; 4 Department of Mathematics and Applied Mathematics, Sefako Makgatho Health Sciences University, Medunsa, Pretoria, South Africa; University of Education, PAKISTAN

## Abstract

This manuscript investigates the convergence behavior of Suzuki’s generalized nonexpansive mappings using the recently introduced Picard–Abbas iteration process. We establish both weak and strong convergence results for the associated fixed-point approximations. To demonstrate the effectiveness of our approach, a numerical example is provided. Furthermore, we generate polynomiographs based on the proposed iteration process and compare them with those produced by existing methods, highlighting the advantages and visual insights offered by our scheme.

## 1 Introduction

Fixed point theory is a versatile and powerful mathematical tool that plays a crucial role in various scientific and engineering disciplines. It is particularly effective for addressing complex nonlinear problems, where conventional analytical methods often prove inefficient or infeasible. The theory has broad applications, including population dynamics in biology [1], market equilibrium models in economics [2], stable strategy profiles in game theory [3], chemical equilibrium analysis in chemistry [4], stability analysis in engineering [4], and algorithm development in artificial intelligence [5]. By leveraging fixed point results, researchers can obtain optimal solutions while minimizing computational costs.

Given the complexity of these applications, standard analytical techniques are often either computationally expensive or incapable of providing exact solutions. Fixed point theory offers a powerful alternative by proving the existence of solutions and furnishing constructive methods to approximate them. A fundamental result in this field is Banach’s Contraction Principle (BCP) [6], which asserts that any contraction operator on a closed subset of a Banach space has a unique fixed point. Moreover, this fixed point can be effectively approximated using the Picard iteration method. This result forms a cornerstone for establishing the existence and approximation of solutions in a wide range of applied problems.

To formally define a contraction mapping, let 𝒢 be a nonempty subset of a Banach space 𝕍. A self-mapping 𝒴:𝒢→𝒢 is said to be a contraction mapping if there exists a constant ζ∈[0,1) such that for all p,x∈𝒢, the following inequality holds:

‖𝒴p−𝒴x‖≤ζ‖p−x‖. (1)

When ζ=1, the mapping is said to be nonexpansive. Furthermore, a point k∈𝒢 is called a fixed point of 𝒴 if 𝒴(k)=k. Throughout this paper, Fix(𝒴) will denote the set of all fixed points of 𝒴. The mapping 𝒴 is said to be quasi-nonexpansive if:

‖𝒴p−k‖≤‖p−k‖ (2)

for all p∈𝒢, and k∈Fix(𝒴).

Over time, various generalizations of contraction mappings have been proposed. One such extension is the class of nonexpansive mappings, introduced independently by Browder [7], Gohde [8], and Kirk [9]. To establish fixed point results for nonexpansive mappings, certain structural conditions such as closedness, boundedness, and uniform convexity are typically required [10]. Suzuki made a significant advancement in this direction [11], who proposed a generalization termed condition (*C*), characterizing a class of mappings now referred to as Suzuki’s generalized nonexpansive mappings. A mapping 𝒴:𝒢→𝒢 is said to satisfy condition (*C*) if, for all p,x∈𝒢, the following holds:

$$\frac{1}{2}\|x-𝒴x\|\leq \|x-p\|\Rightarrow \|𝒴x-𝒴p\|\leq \|x-p\|.$$ (3)

Suzuki demonstrated that this class of mappings forms a broader category than quasi-nonexpansive mappings but is not as general as the class of nonexpansive mappings. Specifically, while every nonexpansive mapping satisfies condition (*C*), the converse does not necessarily hold. The following example illustrates this distinction.

**Example 1.1** ([11]). Define a mapping 𝒴:[0,3]→[0,3] by

$$𝒴(p)=\left\{ \begin{array}{cc} 1, & \text{if }p=3,\\ 0, & \text{otherwise.} \end{array}\right.$$ (4)

In this example, 𝒴 satisfies condition (*C*) but is not a nonexpansive mapping.

Determining the fixed points of various classes of nonlinear mappings is a mathematically challenging task. This challenge is compounded by the failure of Picard iteration to converge for nonexpansive mappings in a complete metric space and by the inapplicability of the Banach Contraction Principle to such mappings. Consequently, numerous iterative procedures have been developed to approximate fixed points of these mappings. These methods have been extensively studied in the literature, notably in the works of Mann [12], Ishikawa [13], Noor [14], Abbas and Nazir [15], Sahu et al. [16], Thakur et al. [17], and Eke and Akewe [18], among others.

Let $\left\{\rho_{n}\right\}$, $\left\{\psi_{n}\right\}$, and $\left\{\eta_{n}\right\}$ be sequences in (0,1), where n∈ℕ. The iteration scheme introduced by Noor [14] is recognized as the first three-step iteration process. This iteration process generates the sequence {*u*_*n*_} as follows:

$$u_{0}\in 𝒢,\;u_{n+1}=(1-\rho_{n})u_{n}+\rho_{n}𝒴y_{n},\;y_{n}=(1-\psi_{n})u_{n}+\psi_{n}𝒴w_{n},\;w_{n}=(1-\eta_{n})u_{n}+\eta_{n}𝒴u_{n}.$$ (5)

Abbas and Nazir proposed a faster iteration process than the Noor iteration, known as the Abbas iteration process [15], which generates the sequence {*u*_*n*_} as follows:

$$u_{0}\in 𝒢,\;u_{n+1}=(1-\rho_{n})𝒴y_{n}+\rho_{n}𝒴w_{n},\;y_{n}=(1-\psi_{n})𝒴u_{n}+\psi_{n}𝒴w_{n},\;w_{n}=(1-\eta_{n})u_{n}+\eta_{n}𝒴u_{n}.$$ (6)

Thakur et al. [17] introduced the following iteration process for approximating the fixed point of nonexpansive mappings:

$$u_{0}\in 𝒢,\;u_{n+1}=(1-\rho_{n})𝒴u_{n}+\rho_{n}𝒴y_{n},\;y_{n}=(1-\psi_{n})w_{n}+\psi_{n}𝒴w_{n},\;w_{n}=(1-\eta_{n})u_{n}+\eta_{n}𝒴u_{n}.$$ (7)

Sahu et al. [16] proposed a new three-step iteration process to approximate fixed points of nonexpansive mappings, generating the sequence {*u*_*n*_} as follows:

$$u_{0}\in 𝒢,\;u_{n+1}=(1-\rho_{n})𝒴w_{n}+\rho_{n}𝒴y_{n},\;y_{n}=(1-\psi_{n})w_{n}+\psi_{n}𝒴w_{n},\;w_{n}=(1-\eta_{n})u_{n}+\eta_{n}𝒴u_{n}.$$ (8)

Eke and Akewe proposed a four-step iteration process, called the Picard–Noor iteration, which generates the sequence {*u*_*n*_} as follows [18]:

$$u_{0}\in 𝒢,\;u_{n+1}=𝒴z_{n},\;z_{n}=(1-\rho_{n})u_{n}+\rho_{n}𝒴y_{n},\;y_{n}=(1-\psi_{n})u_{n}+\psi_{n}𝒴w_{n},\;w_{n}=(1-\eta_{n})u_{n}+\eta_{n}𝒴u_{n}.$$ (9)

A recent contribution by Manbhalang and Naveen [19] introduced the Picard–Abbas iteration process and established both weak and strong convergence results for contraction mappings. The Picard–Abbas iteration process is defined as follows:

$$u_{0}\in 𝒢,\;u_{n+1}=𝒴z_{n},\;z_{n}=(1-\rho_{n})𝒴y_{n}+\rho_{n}𝒴w_{n},\;y_{n}=(1-\psi_{n})𝒴u_{n}+\psi_{n}𝒴w_{n},\;w_{n}=(1-\eta_{n})u_{n}+\eta_{n}𝒴u_{n}.$$ (10)

In recent years, Suzuki’s generalized nonexpansive mappings have attracted considerable attention across various mathematical disciplines, leading to significant progress in fixed-point theory (see [20–23]). These mappings are particularly valuable for the development and analysis of iterative methods due to their rich structural properties and nuanced convergence behavior.

In this work, we investigate the convergence properties of Suzuki’s generalized nonexpansive mappings using the Picard–Abbas iteration process. Our study not only extends existing results but also offers a comparative perspective by analyzing the performance of several established iteration schemes, including those by Noor, Abbas, Thakur, Sahu, and the Picard–Noor iteration processes. To complement our theoretical findings, we present a new numerical example and employ polynomiography—a modern digital visualization technique—to depict the convergence behavior of the various iteration processes. This visual approach enhances the interpretability of the results and facilitates a deeper understanding of their dynamics.

The structure of this paper is as follows. Sect 2 introduces key definitions and fundamental lemmas. In Sect 3, we establish fixed-point results for the proposed iteration process. In Sect 4, we provide a numerical example to demonstrate the effectiveness of the scheme. Sect 5 illustrates the iteration process using visualizations generated through polynomiography. Sect 6 concludes the paper with final remarks.

## 2 Preliminaries

The following basic results are key to proving our main result.

**Definition 2.1** ([10]). A Banach space 𝕍 is said to be a uniformly convex Banach space (UCBS) if, for all δ∈(0,2], there exists ς>0 such that

$$\left(\begin{array}{c} \|q_{1}\|\leq 1,\\ \|q_{2}\|\leq 1,\\ \|q_{1}-q_{2}\|>\delta , \end{array}\right\}\Rightarrow ||\frac{q_{1}+q_{2}}{2}||\leq \varsigma ,\;\text{for all }q_{1},q_{2}\in V.$$ (11)

**Definition 2.2.** Let 𝒢 be a convex and closed subset of a Banach space 𝕍, and let {*u*_*n*_} be a bounded sequence in 𝕍. For any u∈𝒢, the asymptotic radius of the sequence {*u*_*n*_} with respect to 𝒢 is defined by


$$r(𝒢,\{u_{n}\})=\inf\left\{\underset{n\to \infty }{lim sup}\|u_{n}-u\|:u\in 𝒢\right\},$$

and the asymptotic center of {*u*_*n*_} with respect to 𝒢 as


$$A(𝒢,\{u_{n}\})=\left\{u\in 𝒢:\underset{n\to \infty }{lim sup}\|u_{n}-u\|=r(𝒢,\{u_{n}\})\right\}.$$

**Definition 2.3** ([24]). A Banach space 𝕍 is said to have Opial’s property if, for every sequence {*u*_*n*_} in 𝕍 that converges weakly to some k∈𝕍 (i.e., $u_{n}⇀k$), the following inequality holds:


$$\underset{n\to \infty }{lim inf}\|u_{n}-k\|<\underset{n\to \infty }{lim inf}\|u_{n}-t\|$$

for all t∈𝕍 with t≠k.

**Proposition 2.4** ([11]). Let 𝒢≠∅ be a subset of a Banach space 𝕍 and let 𝒴:𝒢→𝒢 be a mapping:

*(a)* *If* 𝒴 *is nonexpansive, then* 𝒴 *satisfies condition (C).*
*(b)* *Any mapping that satisfies condition (C) and has a fixed point is quasi-nonexpansive.*
*(c)* *If* 𝒴 *fulfills condition (C), then*
  ‖p−𝒴x‖≤3‖p−𝒴p‖+‖p−x‖,∀ p,x∈𝒢.

**Lemma 2.5** ([11]). *Let* 𝒢≠∅ *be a subset of a Banach space* 𝕍 *equipped with Opial’s property. Let* 𝒴:𝒢→𝒢 *be a mapping satisfying condition (C). If* {*u*_*n*_} *converges weakly to k and* $\|𝒴u_{n}-u_{n}\|=0$*, then* 𝒴k=k.

The concept of condition (*I*), originally introduced by Senter and Dotson [25], serves as an alternative approach for demonstrating the strong convergence of certain iterative processes in non-compact domains.

**Definition 2.6.** Let 𝒢≠∅ be a subset of a Banach space 𝕍 and let 𝒴 be a self-mapping defined on 𝒢. The mapping 𝒴 is said to satisfy condition (*I*) if there exists a non-decreasing function g:[0,∞)→[0,∞) with g(0)=0 and *g*(*u*) > 0 for all *u* > 0, such that


‖p−𝒴p‖≥g(d(p,Fix(𝒴)))for all p∈𝒢,

where d(p,Fix(𝒴))=inf{‖p−q‖:q∈Fix(𝒴)}.

**Lemma 2.7** ([11]). *Let* 𝒢 *be a weakly compact convex subset of a UCBS* 𝕍*, and let* 𝒴 *be a self-map on* 𝒢*. Assume that* 𝒴 *satisfies condition (C), then* 𝒴 *has a fixed point.*

**Lemma 2.8**([26]). *Suppose* 𝕍 *is a UCBS and for all* n≥1*, we have* $0<a\leq \lambda_{n}\leq b<1$*. Suppose* {*u*_*n*_} *and* $\left\{v_{n}\right\}$ *are two sequences in* 𝕍 *satisfying* $\underset{n\to \infty }{lim sup}\|u_{n}\|\leq \delta$, $\underset{n\to \infty }{lim sup}\|v_{n}\|\leq \delta$ *and* $\lim_{n\to \infty }\|(1-\lambda_{n})u_{n}+\lambda_{n}v_{n}\|=\delta$ *holds for some* δ≥0*. Then,* $\lim_{n\to \infty }\|u_{n}-v_{n}\|=0$.

## 3 Main Results

This section presents convergence results for mappings that satisfy condition (*C*), utilizing the Picard–Abbas iteration process.

**Lemma 3.1.** *Let* 𝒢≠∅ *be a closed and convex subset of a UCBS* 𝕍*. Suppose that* 𝒴:𝒢→𝒢 *is a mapping satisfying condition (C) with* Fix(𝒴)≠∅*. Let* {*u*_*n*_} *be the sequence generated by the Picard–Abbas iteration process* (10). *Then, for any* k∈Fix(𝒴)*, the sequence* {*u*_*n*_} *satisfies*


$$\lim_{n\to \infty }\|u_{n}-k\|=0.$$

*Proof*: Let k∈Fix(𝒴) and u∈𝒢. Since 𝒴 satisfies condition (*C*), by Proposition 2.4(b), we have that 𝒴 is quasi non-expansive mapping, i.e.,


‖𝒴u−k||≤||u−k‖, ∀ u∈𝒢,  k∈Fix(𝒴).

Using (10), we get

$$||w_{n}-k||=\|(1-\eta_{n})u_{n}+\eta_{n}𝒴u_{n}-k\|\;\leq (1-\eta_{n})||u_{n}-k||+\eta_{n}||𝒴u_{n}-k||\;\leq (1-\eta_{n})||u_{n}-k||+\eta_{n}||u_{n}-k||\;=||u_{n}-k||.$$ (12)

And,


$$||y_{n}-k||=||(1-\psi_{n})𝒴u_{n}+\psi_{n}𝒴w_{n}-k||\;=||(1-\psi_{n})(𝒴u_{n}-k)+\psi_{n}(𝒴w_{n}-k)||\;\leq (1-\psi_{n})||𝒴u_{n}-k||+\psi_{n}||𝒴w_{n}-k||\;\leq (1-\psi_{n})||u_{n}-k||+\psi_{n}||w_{n}-k||.$$

Using (12), we have

$$||y_{n}-k||\leq (1-\psi_{n})||u_{n}-k||+\psi_{n}||u_{n}-k||=||u_{n}-k||.$$ (13)

Also


$$||z_{n}-k||=||(1-\rho_{n})𝒴y_{n}+\rho_{n}𝒴w_{n}-k||\;=||(1-\rho_{n})(𝒴y_{n}-k)+\rho_{n}(𝒴w_{n}-k)||\;\leq (1-\rho_{n})||𝒴y_{n}-k||+\rho_{n}||𝒴w_{n}-k||\;\leq (1-\rho_{n})||y_{n}-k||+\rho_{n}||w_{n}-k||.$$

Using (12) and (13), we obtain

$$||z_{n}-k||\leq ||u_{n}-k||.$$ (14)

Similarly,


$$||u_{n+1}-k||=||𝒴z_{n}-k||\leq ||z_{n}-k||.$$

By using (14), we get

$$||u_{n+1}-k||\leq ||u_{n}-k||.$$ (15)

It follows from (12)–(15) that


$$\|u_{n+1}-k\|\leq \|u_{n}-k\|.$$

Hence, the sequence $\left\{\|u_{n}\;-\;k\|\right\}$ is both bounded and non-increasing. Thus, we can conclude that $\lim_{n\to \infty }||u_{n}-k||$ exists for each k∈Fix(𝒴). □

Next, we discuss the existence of a fixed point for mappings satisfying condition (*C*).

**Theorem 3.2.** *Let* 𝕍 *be a UCBS, and let* 𝒢⊆𝕍 *be a nonempty, closed and convex subset. Suppose that* 𝒴:𝒢→𝒢 *is a mapping satisfying condition (C), and let* {*u*_*n*_} *be the sequence generated by the Picard–Abbas iteration process* (10)*. Then,* Fix(𝒴) *is nonempty if and only if the sequence* {*u*_*n*_} *is bounded and* $\lim_{n\to \infty }||u_{n}-𝒴u_{n}||=0$.

*Proof*: Suppose that Fix(𝒴)≠∅ and let k∈Fix(𝒴). By Lemma 3.1, we conclude that the sequence *u*_*n*_ is bounded and that the limit $\lim_{n\to \infty }||u_{n}-k||$ exists and it is finite. Define

$$\xi =\lim_{n\to \infty }||u_{n}-k||.$$ (16)

From Lemma 3.1, we get


$$||w_{n}-k||\leq ||u_{n}-k||.$$

Thus,

$$\underset{n\to \infty }{lim sup}||w_{n}-k||\leq {lim sup}_{n\to \infty }||u_{n}-k||=\xi .$$ (17)

As 𝒴 satisfies condition (*C*), and by Preposition 2.4(b), we get


$$||𝒴u_{n}-k||\leq ||u_{n}-k||.$$

Thus,

$$\underset{n\to \infty }{lim sup}||𝒴u_{n}-k||\leq {lim sup}_{n\to \infty }||u_{n}-k||=\xi .$$ (18)

By Lemma 3.1, we get

$$||u_{n+1}-k||\leq ||w_{n}-k||.$$ (19)

Using (19) and (17), we have

$$\xi \leq \underset{n\to \infty }{lim inf}||w_{n}-k||.$$ (20)

From (17) and (20), we obtain that

$$\xi =\lim_{n\to \infty }||w_{n}-k||.$$ (21)

From Lemma 3.1, one has


$$||w_{n}-k||=\|\eta_{n}(𝒴u_{n}-k)+(1-\eta_{n})(u_{n}-k)\|,$$

so

$$\lim_{n\to \infty }||w_{n}-k||=\lim_{n\to \infty }\|\eta_{n}(𝒴u_{n}-k)+(1-\eta_{n})(u_{n}-k)\|.$$ (22)

Using (21) and (22), we get

$$\xi =\lim_{n\to \infty }||w_{n}-k||=\lim_{n\to \infty }\|\eta_{n}(𝒴u_{n}-k)+(1-\eta_{n})(u_{n}-k)\|.$$ (23)

Using Lemma 2.8 with (16), (18) and (23), we have


$$\lim_{n\to \infty }||𝒴u_{n}-u_{n}||=0.$$

Conversely, suppose that {*u*_*n*_} is bounded and $\lim_{n\to \infty }||𝒴u_{n}-u_{n}||=0$. Let $k\in A(𝒢,\{u_{n}\})$, by Proposition 2.4(c), we obtain


$$r(𝒴k,\{u_{n}\})=\underset{n\to \infty }{lim sup}||u_{n}-𝒴k||\;\leq 3\underset{n\to \infty }{lim sup}||𝒴u_{n}-u_{n}||+\underset{n\to \infty }{lim sup}||u_{n}-k||\;=\underset{n\to \infty }{lim sup}||u_{n}-k||=r(k,\{u_{n}\})=r(A,\{u_{n}\}),$$

which implies that $𝒴k\in A(𝒢,\{u_{n}\})$. As 𝒢 is UCBS, therefore $A(𝒢,\{u_{n}\})$ is a singleton, which means 𝒴k=k. Hence, Fix(𝒴)≠∅. □

Next, we prove weak convergence using Opial’s property.

**Theorem 3.3.** *Let* ∅≠𝒢 *be a closed and convex subset of a UCBS* 𝕍*. Let* 𝒴:𝒢→𝒢 *be a mapping satisfying condition (C) with* Fix(𝒴)≠∅*. Suppose that the space* 𝕍 *satisfies Opial’s condition. If* {*u*_*n*_} *is the sequence generated by the Picard–Abbas iteration process* (10)*, then* {*u*_*n*_} *converges weakly to a fixed point of* 𝒴.

*Proof*: By Theorem 3.2, the sequence {*u*_*n*_} is bounded and satisfies $\lim_{n\to \infty }\|u_{n}\;-\;𝒴u_{n}\|=0$. Since 𝕍 is a uniformly convex Banach space, it is reflexive. Thus, there exists a subsequence $\left\{u_{n_{m}}\right\}$ of {*u*_*n*_} that converges weakly to some $x_{1}\in 𝕍$. By Lemma 2.5, it follows that $x_{1}\in \mathrm{Fix}(𝒴)$.

To show that {*u*_*n*_} converges weakly to *x*_1_, assume, for the sake of contradiction, that it does not. Then there exists another subsequence $\left\{u_{n_{s}}\right\}$ of {*u*_*n*_} such that $\left\{u_{n_{s}}\right\}$ converges weakly to $x_{2}\in 𝕍$ with $x_{2}\neq x_{1}$. Again, by Lemma 2.5, we have $x_{2}\in \mathrm{Fix}(𝒴)$.

Now, by applying Opial’s condition together with Lemma 3.1, we obtain


$$\lim_{n\to \infty }||u_{n}-x_{1}||=\lim_{m\to \infty }||u_{n_{m}}-x_{1}||<\lim_{m\to \infty }||u_{n_{m}}-x_{2}||\;=\lim_{n\to \infty }||u_{n}-x_{2}||=\lim_{s\to \infty }||u_{n_{s}}-x_{2}||\;<\lim_{s\to \infty }||u_{n_{s}}-x_{1}||=\lim_{n\to \infty }||u_{n}-x_{1}||.$$

This contradicts our supposition, so $x_{1}=x_{2}$. Thus, {*u*_*n*_} converges weakly to a point in Fix(𝒴). □

Now, we use the concept of compactness to prove strong convergence.

**Theorem 3.4.** *Let* 𝒴 *be a mapping satisfying condition (C) defined on a nonempty, closed, and compact subset* 𝒢 *of a uniformly convex Banach space* 𝕍*. Let* {*u*_*n*_} *be a sequence generated by* (10)*. Then,* {*u*_*n*_} *converges strongly to a fixed point of* 𝒴.

*Proof*: By using Lemma 2.7, we obtain Fix(𝒴)≠∅. Since Fix(𝒴)≠∅, it follows from Lemma 3.2 that $\lim_{n\to \infty }||𝒴u_{n}\;-\;u_{n}||=0$ . As 𝒢 is given to be compact and closed, there exists a subsequence $\left\{u_{n_{m}}\right\}$ of {*u*_*n*_} in 𝒢 such that it converges strongly to some k∈𝒢, i.e., $\lim_{n_{m}\to \infty }||u_{n_{m}}-k||=0$. Hence, using these facts together with Proposition 2.4(c), we obtain

$$\|u_{n_{m}}-𝒴k\|\leq 3\|𝒴u_{n_{m}}-u_{n_{m}}\|+\|u_{n_{m}}-k\|.$$ (24)

Letting $n_{m}\to \infty$, we obtain $u_{n_{m}}\to 𝒴k$. This implies 𝒴k=k which means that k∈Fix(𝒴). Moreover, Lemma 3.1 implies that the limit $\lim_{n\to \infty }||u_{n}-k||$ exists. Hence, *k* is the strong limit of the sequence {*u*_*n*_}. □

The following theorem proves strong convergence without requiring compactness.

**Theorem 3.5.** *Let* 𝕍 *be a UCBS, and let* 𝒢 *be a nonempty, closed, and convex subset of* 𝒱. *Suppose that* 𝒴:𝒢→𝒢 *is a mapping satisfying condition (C), and let* {*u*_*n*_} *be a sequence generated by* (10)*. Then,* {*u*_*n*_} *converges to a point in* Fix(𝒴) *if and only if*


$$\underset{n\to \infty }{lim inf}d(u_{n},Fix(𝒴))=0.$$

*Proof*: Suppose that the sequence {*u*_*n*_} converges to some k∈Fix(𝒴). Then, by the definition of convergence,


$$\lim_{n\to \infty }\|u_{n}-k\|=0,$$

it follows that


$$\underset{n\to \infty }{lim inf}d(u_{n},Fix(𝒴))=0.$$

Conversely, assume that $\underset{n\to \infty }{lim inf}d(u_{n},Fix(𝒴))=0$. From Lemma 3.1, the limit $\lim_{n\to \infty }\|u_{n}-k\|$ exists, which gives


$$\left\|u_{n+1}-k\|\leq \|u_{n}-k\right\|$$

and this provides

$$d(u_{n+1},Fix(𝒴))\leq d(u_{n},Fix(𝒴)).$$ (25)

Therefore $\left\{d(u_{n},Fix(𝒴))\right\}$ constitutes a decreasing sequence that is bounded below by zero, so it may be obtained that $\lim_{n\to \infty }\{d(u_{n},Fix(𝒴))\}$ exists. Since $\underset{n\to \infty }{lim inf}d(u_{n},Fix(𝒴))=0$, so $d(u_{n},Fix(𝒴))=0$. We now show that {*u*_*n*_} is a Cauchy sequence in 𝒢.

Since $d(u_{n},Fix(𝒴))=0$, for any ϵ>0, there exists an integer $m_{0}\in \mathbb{N}$ such that for all $n\geq m_{0}$,


$$d(u_{n},Fix(𝒴))<\frac{\epsilon }{2}.$$

Especially,


$$\inf\{\|u_{n}-k\|:k\in Fix(𝒴)\}<\frac{\epsilon }{2}.$$

Thus, we can choose some s∈Fix(𝒴) such that

$$\|u_{n_{0}}-s\|<\frac{\epsilon }{2}.$$ (26)

For any $n,m\geq n_{0}$, applying the triangle inequality, we obtain


$$\|u_{n+m}-u_{n}\|\leq \|u_{n+m}-s\|+\|u_{n}-s\|.$$

Since both terms on the right-hand side are bounded by $\left\|u_{n_{0}}-s\right\|$, it follows that


$$\|u_{n+m}-u_{n}\|\leq 2\|u_{n_{0}}-s\|,$$

which implies that {*u*_*n*_} is a Cauchy sequence in 𝒢.

Since 𝒢 is a closed subset of the Banach space 𝕍, then {*u*_*n*_} is convereges in 𝒢. Consider $\lim_{n\to \infty }u_{n}=p$ for any p∈𝒢. Applying $\lim_{n\to \infty }\|u_{n}-p\|=0$, one obtains


$$\|p-𝒴p\|\leq \|p-u_{n}\|+\|u_{n}-𝒴u_{n}\|+\|𝒴u_{n}-𝒴p\|\;\leq \|p-u_{n}\|+\|u_{n}-𝒴u_{n}\|+\|u_{n}-p\|.\;\to 0\;as\;n\to \infty$$

Thus, p=𝒴p, hence p∈Fix(𝒴). □

We now use condition (*I*) to prove the strong convergence of the Picard-Abbas iteration process. This condition imposes additional constraints that strengthen convergence, especially when generalized non-expansiveness alone is not enough. It ensures norm convergence by linking the iterative sequence to a fixed point and guaranteeing that the distance between successive iterates gradually decreases.

**Theorem 3.6.** *Let* 𝒢 *be a closed and convex subset of UCBS* 𝕍*. Suppose that* 𝒴:𝒢→𝒢 *is a mapping satisfying condition (I), and let* {*u*_*n*_} *be a sequence generated by* (10)*. Then, the sequence* {*u*_*n*_} *converges strongly to a fixed point of* 𝒴.

*Proof*: From (25), one can get $\lim_{n\to \infty }d(u_{n},Fix(𝒴))$ exist and by Theorem 3.2, we obtain

$$\lim_{n\to \infty }||u_{n}-𝒴u_{n}||=0.$$ (27)

From condition (*I*) and (27), we have


$$\lim_{n\to \infty }g(d(u_{n},Fix(𝒴)))\leq \lim_{n\to \infty }||u_{n}-𝒴u_{n}||=0.$$

Therefore, $\lim_{n\to \infty }g(d(u_{n},Fix(𝒴)))=0.$ Since *g* is a nondecreasing function with g(0)=0, *g*(*u*) > 0 for each *u* > 0, therefore we have


$$\lim_{n\to \infty }d(u_{n},Fix(𝒴))=0.$$

Hence, all conditions of Theorem 3.5 are satisfied, therefore, {*u*_*n*_} converges strongly to a fixed point of 𝒴. □

## 4 Numerical example

This section introduces a novel numerical example to demonstrate the convergence properties of mappings satisfying condition (*C*), as analyzed through the Picard–Abbas iteration process.

**Example 4.1.** Define 𝒴:[0,1]→[0,1] such that

$$𝒴p=\left\{ \begin{array}{cc} 1-p, & \text{ if }p\in [0,\frac{1}{7}),\\ \frac{p+4}{5}, & \text{ if }p\in [\frac{1}{7},1]. \end{array}\right.$$ (28)

First, we show that the given mapping is not a nonexpansive mapping. For $p=\frac{7}{55}$ and $x=\frac{1}{7}$, we obtain


$$𝒴p=\frac{48}{55},$$

$$𝒴x=\frac{29}{35},$$

$$|p-x|=\left|\frac{7}{55}-\frac{1}{7}\right|=\frac{6}{385}.$$

Now,


$$|𝒴p-𝒴x|=\left|\frac{48}{55}-\frac{29}{35}\right|=\frac{17}{385}.$$

We can notice that


$$|𝒴p-𝒴x|=\frac{17}{385}>\frac{6}{385}=|p-x|.$$

Therefore, the mapping given in (28) is not a nonexpansive mapping.

Now, we prove that 𝒴 given in (28) satisfies condition (*C*).

1. When $p\in \left[0,\frac{1}{7}\right)$, then 𝒴p=1−p, and
  $$\frac{1}{2}|p-𝒴p|=\frac{1}{2}|p-(1-p)|=\frac{1}{2}(|1-2p|).$$
  For $\frac{\left|p-𝒴p\right|}{2}\leq |p-x|$, we must have $\frac{1-2p}{2}\leq x-p$. Thus,
  $$\frac{1-2p}{2}+p\leq x\Rightarrow x\in \left[\frac{1}{2},1\right].$$
  So, $p\in \left[0,\frac{1}{7}\right)$ and $x\in \left[\frac{1}{2},1\right]$ implies that
  $$𝒴p=1-p,\;𝒴x=\frac{x+4}{5}.$$
  Now,
  $$|𝒴p-𝒴x|=\left|1-p-\frac{x+4}{5}\right|=\left|\frac{5p+x-1}{5}\right|\leq |p-x|.$$
  Thus,
  $$\frac{1}{2}|p-𝒴p|\leq |p-x|\Rightarrow |𝒴p-𝒴x|\leq |p-x|,$$
  which implies that 𝒴 satisfies condition (*C*).
2. When $p\in \left[\frac{1}{7},1\right]$ then $𝒴p=\frac{p+4}{5}$, and
  $$\frac{1}{2}|p-𝒴p|=\frac{1}{2}\left|p-\frac{p+4}{5}\right|=\frac{4-4p}{10}\in \left[0,\frac{12}{35}\right].$$
  For $\frac{\left|p-𝒴p\right|}{2}\leq |p-x|$, we must have $\frac{4-4p}{10}\leq |p-x|$. Now, we have two cases:
  (i) *x* > *p*. In this case, we have $\frac{4-4p}{10}\leq x-p$. Thus,
    $$x\geq \frac{6p+4}{10}.$$
    Which implies that $x\in \left[\frac{17}{35},1\right]\subset \left[\frac{1}{7},1\right]$.
    Therefore, $𝒴p=\frac{p+4}{5},\;𝒴x=\frac{x+4}{5}$. So,
    $$|𝒴p-𝒴x|=\left|\frac{p+4}{5}-\frac{x+4}{5}\right|=\left|\frac{p+x}{5}\right|\leq |p+x|,$$
    which means that $\frac{1}{2}|p-𝒴p|\leq |p-x|$ implies |𝒴p−𝒴x|≤|p−x|, so 𝒴 satisfies condition (*C*).
  (ii) *p*>*x*. In this case, we have $\frac{4-4p}{10}\leq p-x$. Thus,
    $$x\leq \frac{14p-4}{10}.$$
    Which implies that $x\in \left[-\frac{1}{5},1\right]$. As x∈[0,1], we get
    $$\frac{10x+4}{14}\leq p\Rightarrow p\in \left[\frac{2}{7},1\right].$$
    Now, let $p\in [\frac{2}{7},1]$ and $x\in [\frac{1}{7},1]$. As $x\in [\frac{1}{7},1]$ is previously discussed in (i), therefore, now, working for $x\in [0,\frac{1}{7})$ and $p\in [\frac{2}{7},1]$, we have $𝒴p=\frac{p+4}{5}$, 𝒴x=1−x. Thus,
    $$|𝒴p-𝒴x|=\left|\frac{p+4}{5}-(1-x)\right|=\left|\frac{5x+p-1}{5}\right|.$$
    We first suppose that $p\in [\frac{2}{7},\frac{1}{2}]$ and $x\in [0,\frac{1}{7})$, then
    $$\left|\frac{5x+p-1}{5}\right|=\frac{3}{70}<\frac{5}{14}=|p-x|.$$
    Which implies $\frac{1}{2}|p-𝒴p|\leq |p-x|\Rightarrow |𝒴p-𝒴x|\leq |p-x|$. Now, let assume that $p\in [\frac{1}{2},1]$ and $x\in [0,\frac{1}{7})$, then
    $$\left|\frac{5x+p-1}{5}\right|=\frac{1}{7}<\frac{6}{7}=|p-x|,$$
    which shows that
    $$\frac{1}{2}|p-𝒴p|\leq |p-x|\Rightarrow |𝒴p-𝒴x\|\leq |p-x|.$$
    Thus, $\frac{1}{2}|p\;-\;𝒴p|\leq |p\;-\;x|\Rightarrow |𝒴p\;-\;𝒴x|\leq |p\;-\;x|$, which shows that 𝒴 satisfies condition (*C*).
    Hence, it is established that 𝒴 is a Suzuki generalized nonexpansive mapping.

**Fig 1:**
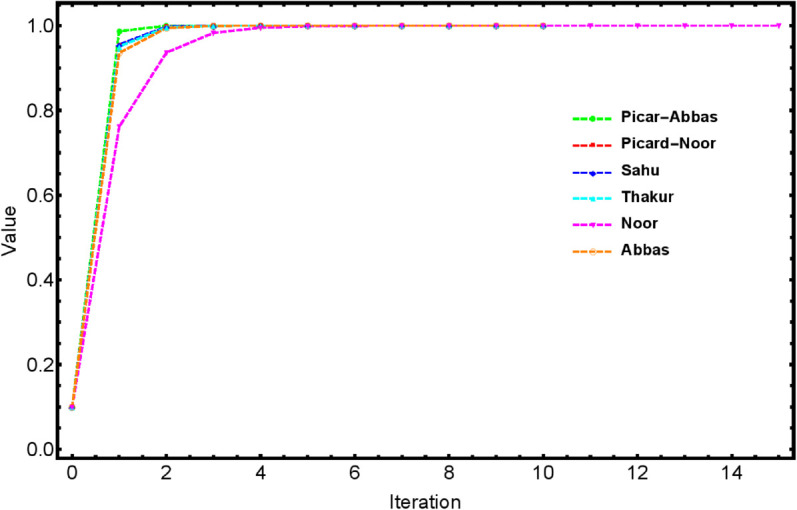
Convergence behavior of Picard–Abbas, Noor, Abbas, Thakur, Sahu and Picard–Noor iteration processes corresponding to Table 1.

To illustrate the faster convergence of the proposed Picard–Abbas iteration process (10), we compare it against the Noor, Abbas, Thakur, Sahu, and Picard–Noor iteration methods. The selected parameters are $\rho_{n}=0.80$, $\psi_{n}=0.65$, and $\eta_{n}=0.65$, with the stopping criterion defined as $||u_{n}\;-\;u_{n+1}||<10^{-8}$ and the initial point *u*_0_ = 0.1. The corresponding results are presented in Fig 1 and Table 1.

**Table 1:** Iterates produced by various iteration processes for the mapping 𝒴 given in (28) and the starting point $u_{0}$ = 0.1.

*n*	Picard–Abbas	Sahu	Picard–Noor	Thakur	Abbas	Noor
	iteration	iteration	iteration	iteration	iteration	iteration
0	0.1	0.1	0.1	0.1	0.1	0.1
1	0.98716480	0.95561600	0.95233920	0.95081600	0.93582400	0.76169600
2	0.99978926	0.99751166	0.99746460	0.99621952	0.99473141	0.93661495
3	0.99999654	0.99986049	0.99986512	0.99970942	0.99956747	0.98314059
4	0.99999994	0.99999218	0.99999283	0.99997766	0.99996449	0.99551567
5	1	0.99999956	0.99999962	0.99999828	0.99999708	0.99880724
6	1	0.99999998	0.99999998	0.99999987	0.99999976	0.99968274
7	1	1	1	0.99999999	0.99999998	0.99991562
8	1	1	1	1	1	0.99997755
9	1	1	1	1	1	0.99999403
10	1	1	1	1	1	0.99999841
11	1	1	1	1	1	0.99999958
12	1	1	1	1	1	0.99999989
13	1	1	1	1	1	0.99999997
14	1	1	1	1	1	0.99999999
15	1	1	1	1	1	1

The findings indicate that, after the first iteration, the value obtained by the Picard–Abbas iteration process (0.98716480) is the closest to the fixed point 1 among all compared methods. As shown in Table 1, each iteration method converges at a different rate. The proposed Picard–Abbas iteration is the fastest, reaching the fixed point in 5 iterations. The Sahu and Picard–Noor iteration methods require 6 iterations to converge. The Thakur and Abbas processes exhibit similar convergence behavior. In contrast, the Noor iteration method shows the slowest convergence, taking 15 iterations to reach the fixed point.

## 5 Comparison via polynomiography

Mathematician and computer scientist Bahman Kalantari introduced polynomiography, a digital art form and visual analytic technique for exploring root-finding problems [27,28]. Although related concepts, such as basins of attraction, dynamical planes, and speed of convergence, had appeared earlier in the literature, Kalantari was the first to consolidate these ideas under a unified framework. He defined polynomiography as the art and science of visualizing the approximation of the zeros of complex polynomials through iterative functions, referring to the resulting images as polynomiographs. Various types of iteration processes have since been compared and analyzed using polynomiographic techniques (see [29–33]).

The general procedure for generating polynomiographs is outlined in Algorithm 1. Color assignment within this algorithm can follow various approaches; in this study, we adopt a method that integrates basins of attraction with convergence speed [34]. Each root of the polynomial 𝒲 is assigned a distinct non-black color, while points that do not converge are marked in black. For each initial point *u*_0_ in the region *A*, the iterative method *I* is applied for up to *K* iterations. If convergence occurs in fewer than *K* steps, we determine the root closest to the resulting point *u*_*n*_ and assign its corresponding color to *u*_0_. The brightness of the color reflects the speed of convergence: lighter shades indicate faster convergence, while darker shades represent slower convergence. If no convergence is achieved within *K* iterations, *u*_0_ is colored black. This scheme effectively visualizes both the destination of convergence (via color) and the convergence rate (via shading), providing intuitive insights into the behavior of the iterative process.

**Algorithm 1. Creation of a polynomiograpgh.**



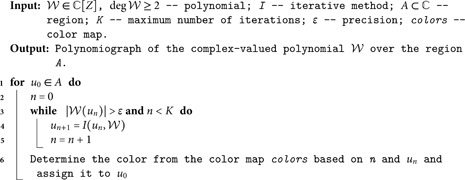



One well-known root-finding algorithm is the Newton’s iteration method, also known as the Newton–Raphson method. Its definition is:

$$u_{n+1}=u_{n}-\frac{𝒲(u_{n})}{{𝒲}^{\prime }(u_{n})},$$ (29)

where $u_{0}\in \mathbb{C}$ is the starting point and 𝒲 is a polynomial with complex coefficients. We can write (29) in terms of a fixed point iteration process as follows:

$$u_{n+1}=𝒴(u_{n}),$$ (30)

where $𝒴(u)=u\;-\;\frac{𝒲(u)}{{𝒲}^{\prime }(u)}$. Thus, this is the Picard iteration. If the iteration process (30) converges to any fixed point x∈ℂ of 𝒴, then one has

$$x=𝒴(x)=x-\frac{𝒲(x)}{{𝒲}^{\prime }(x)}.$$ (31)

Thus, $\frac{𝒲(x)}{{𝒲}^{\prime }(x)}=0$, which means that *x* is a root of 𝒲. Finding the fixed points of 𝒴 is therefore equal to solving the problem of finding the roots of 𝒲. This enables us to use various fixed point iteration processes for 𝒴, such as the suggested Picard–Abbas iteration.

In the considered example, we use three sets of iterations’ parameters

- $\rho_{n}=0.03$, $\psi_{n}=0.06$, $\eta_{n}=0.06$;
- $\rho_{n}=0.6$, $\psi_{n}=0.6$, $\eta_{n}=0.6$;
- $\rho_{n}=0.9$, $\psi_{n}=0.9$, $\eta_{n}=0.9$.

For each of the three sets of iteration parameters, we generated polynomiographs of the polynomial $𝒲(u)=u^{6}\;-\;1$, which has six roots: –1.0, –0.5–0.866025i, −0.5+0.866025i, 0.5–0.866025i, 0.5+0.86602540i, and 1.0. The iteration schemes used include Picard–Abbas, Sahu, Abbas, Thakur, Noor, and Picard–Noor methods. The parameters for polynomiograph generation were: region *A* = *[*−2,2*]*^2^, maximum number of iterations *K* = 45, and ε=0.001. Additionally, for each polynomiograph, we computed the Average Number of Iterations (ANI) as proposed in [35].

**Fig 2:**
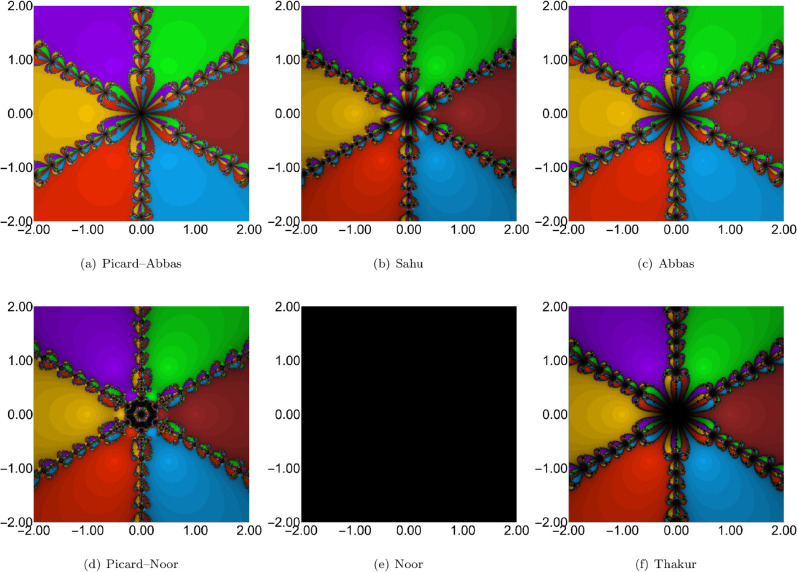
Comparison of polynomiographs obtained from different iteration processes with parameters $\rho_{n}$ = 0.06, $\psi_{n}$ = 0.06, $\eta_{n}$ = 0.06.

The polynomiographs generated for the first set of parameter values are shown in Fig 2. Distinct convergence patterns are observed for the Picard–Abbas, Sahu, Abbas, Thakur, Noor, and Picard–Noor iteration processes. Visual inspection indicates that the proposed Picard–Abbas iteration exhibits the fastest convergence, followed by Abbas, Picard–Noor, Sahu, Thakur, and Noor. Notably, for the Noor iteration, no points within the considered region converged to any root, resulting in a completely black polynomiograph. ANI values in Table 2 corroborate these findings: Picard–Abbas (4.156), Abbas (6.110), Picard–Noor (8.392), Sahu (8.609), and Thakur (10.786).

**Table 2:** ANI values of the polynomiographs given in Figs 2–4.

Iteration	$\rho_{n}=\psi_{n}=\eta_{n}=0.06$	$\rho_{n}=\psi_{n}=\eta_{n}=0.6$	$\rho_{n}=\psi_{n}=\eta_{n}=0.9$
Picard–Abbas	4.156	3.746	3.817
Sahu	8.609	5.216	4.388
Abbas	6.110	5.390	5.370
Picard–Noor	8.392	4.778	3.720
Noor	45	12.068	6.223
Thakur	10.786	6.078	4.738

**Fig 3:**
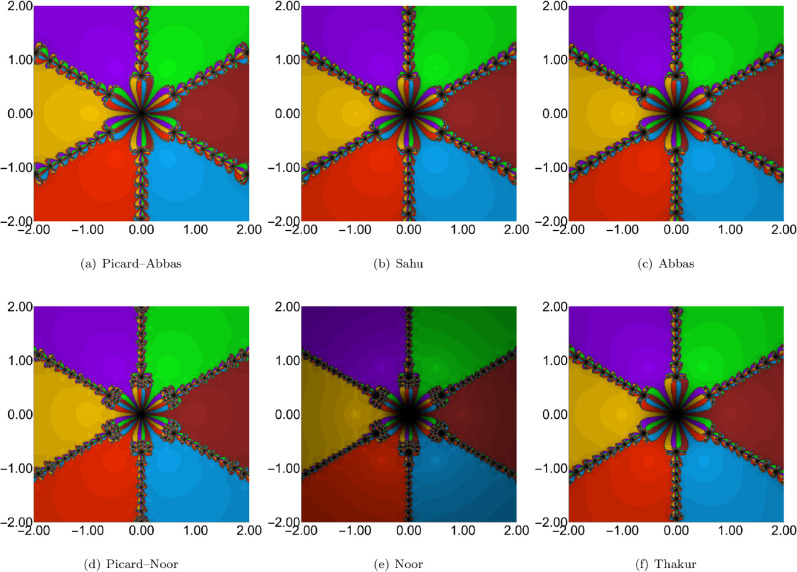
Comparison of polynomiographs obtained from different iteration processes with parameters $\rho_{n}$ = 0.6, $\psi_{n}$ = 0.6, $\eta_{n}$ = 0.6.

**Fig 4:**
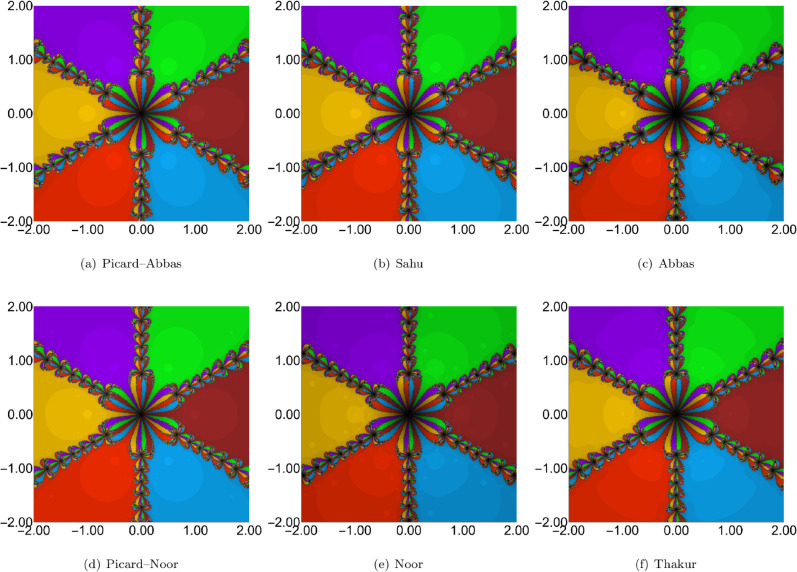
Comparison of polynomiographs obtained from different iteration processes with parameters $\rho_{n}$ = 0.9, $\psi_{n}$ = 0.9, and $\eta_{n}$ = 0.9.

The polynomiographs for the parameter settings $\rho_{n}=0.6$, $\psi_{n}=0.6$, and $\eta_{n}=0.6$ are shown in Fig 3. The results show that the Noor iteration exhibits the slowest convergence speed, with the highest ANI value of 12.068. Among the iterations studied, the Picard–Abbas method achieves the fastest convergence, yielding the lowest ANI value of 3.746. In terms of convergence speed, the Picard–Noor iteration ranks second with an ANI of 4.778, followed by the Sahu (5.216), Abbas (5.390), and Thakur (6.078) iterations.

The third configuration employs high values for the iteration parameters. Similar to the previous cases, the Noor iteration exhibits the slowest convergence, as shown in Fig 4. In contrast, the Picard–Abbas iteration once again achieves the fastest convergence. Interestingly, the high-parameter setting leads to faster convergence across all methods, requiring fewer iterations to reach the polynomial’s roots. The ANI values corresponding to this configuration are presented in Table 2. The Picard–Noor iteration yields the lowest ANI value of 3.720, followed closely by the Picard–Abbas iteration with an ANI of 3.817.

## 6 Conclusion

Our analysis of Suzuki mappings using the Picard–Abbas iteration process demonstrates its enhanced convergence performance. The numerical results in Table 1 confirm its efficiency relative to several established methods, including those of Noor, Sahu, Thakur, Abbas, and Picard–Noor. Furthermore, visualizations generated through polynomiography provide additional insight into the convergence behavior, highlighting the iteration’s faster convergence rate. Collectively, these findings suggest that the Picard–Abbas process is a robust and effective tool for solving fixed-point problems, with promising potential for broader applications in mathematical and computational contexts.
